# Applying an evolutionary mismatch framework to understand disease susceptibility

**DOI:** 10.1371/journal.pbio.3002311

**Published:** 2023-09-11

**Authors:** Amanda J. Lea, Andrew G. Clark, Andrew W. Dahl, Orrin Devinsky, Angela R. Garcia, Christopher D. Golden, Joseph Kamau, Thomas S. Kraft, Yvonne A. L. Lim, Dino J. Martins, Donald Mogoi, Päivi Pajukanta, George H. Perry, Herman Pontzer, Benjamin C. Trumble, Samuel S. Urlacher, Vivek V. Venkataraman, Ian J. Wallace, Michael Gurven, Daniel E. Lieberman, Julien F. Ayroles

**Affiliations:** 1 Department of Biological Sciences, Vanderbilt University, Nashville, Tennessee, United States of America; 2 Department of Molecular Biology & Genetics, Cornell University, Ithaca, New York, United States of America; 3 Department of Medicine, University of Chicago, Chicago, Illinois, United States of America; 4 Department of Neurology, NYU Langone Comprehensive Epilepsy Center, NYU Grossman School of Medicine, New York, New York, United States of America; 5 Department of Anthropology, Stanford University, Stanford, California, United States of America; 6 Department of Nutrition, Harvard T H Chan School of Public Health, Boston, Massachusetts, United States of America; 7 One Health Centre, Institute of Primate Research, Karen, Nairobi, Kenya; 8 Department of Anthropology, University of Utah, Salt Lake City, Utah, United States of America; 9 Department of Parasitology, Faculty of Medicine, Universiti Malaya, Kuala Lumpur, Malaysia; 10 Turkana Basin Institute, Stony Brook University, Stony Brook, New York, United States of America; 11 Department of Medical Services and Public Health, Ministry of Health Laikipia County, Nanyuki, Kenya; 12 Department of Human Genetics, David Geffen School of Medicine at UCLA, University of California Los Angeles, Los Angeles, California, United States of America; 13 Departments of Anthropology and Biology, The Pennsylvania State University, University Park, Pennsylvania, United States of America; 14 Department of Evolutionary Anthropology, Duke University, Durham, North Carolina, United States of America; 15 Duke Global Health Institute, Duke University, Durham, North Carolina, United States of America; 16 School of Human Evolution and Social Change, Arizona State University, Tempe, Arizona, United States of America; 17 Center for Evolution and Medicine, Arizona State University, Tempe, Arizona, United States of America; 18 Department of Anthropology, Baylor University, Waco, Texas, United States of America; 19 Department of Anthropology and Archaeology, University of Calgary, Calgary, Alberta, Canada; 20 Department of Anthropology, University of New Mexico, Albuquerque, New Mexico, United States of America; 21 Department of Anthropology, University of California Santa Barbara, Santa Barbara, California, United States of America; 22 Department of Human Evolutionary Biology, Harvard University, Cambridge, Massachusetts, United States of America; 23 Lewis-Sigler Institute for Integrative Genomics, Princeton University, Princeton, New Jersey, United States of America; 24 Department of Ecology and Evolutionary Biology, Princeton University, Princeton, New Jersey, United States of America

## Abstract

Noncommunicable diseases (NCDs) are on the rise worldwide. Obesity, cardiovascular disease, and type 2 diabetes are among a long list of “lifestyle” diseases that were rare throughout human history but are now common. The evolutionary mismatch hypothesis posits that humans evolved in environments that radically differ from those we currently experience; consequently, traits that were once advantageous may now be “mismatched” and disease causing. At the genetic level, this hypothesis predicts that loci with a history of selection will exhibit “genotype by environment” (GxE) interactions, with different health effects in “ancestral” versus “modern” environments. To identify such loci, we advocate for combining genomic tools in partnership with subsistence-level groups experiencing rapid lifestyle change. In these populations, comparisons of individuals falling on opposite extremes of the “matched” to “mismatched” spectrum are uniquely possible. More broadly, the work we propose will inform our understanding of environmental and genetic risk factors for NCDs across diverse ancestries and cultures.

## Abstract 2

Humans evolved in environments that radically differ from those we currently experience; thus, traits that were once advantageous may now be “mismatched” and disease-causing. In this Essay, the authors advocate for combining genomic tools with partnerships with subsistence-level groups experiencing rapid lifestyle change to identify genetic loci associated with disease risk.

## Introduction

**Fig 1:**
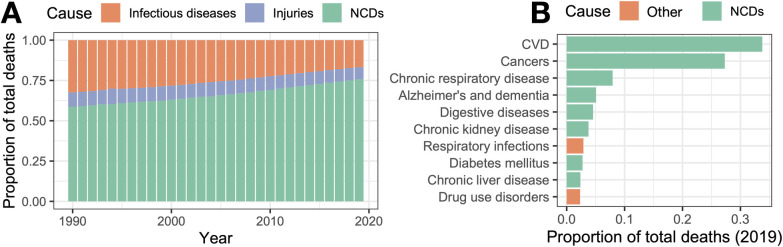
Noncommunicable diseases are the leading cause of death worldwide. (**A**) Proportion of worldwide deaths attributable to noncommunicable diseases (NCDs), communicable or infectious diseases, and injuries through time. (**B**) Proportion of deaths within the USA in 2019, broken down by the top 10 causes of death. NCDs are highlighted in green. For both panels, data were sourced from ourworldindata.org and represent all ages.

Noncommunicable diseases (NCDs) such as cardiovascular disease (CVD), type 2 diabetes, and Alzheimer’s disease are among the leading causes of death worldwide (Fig 1). NCDs are often difficult to prevent and treat, because they result from complex and poorly understood interactions between a person’s genetic makeup and their environment. For example, CVD has a heritability of 40% to 50%, with dozens of loci now mapped through genome-wide association studies [1–3]. However, when tallied together in an additive framework, these loci explain only a small fraction of the heritable genetic effect. This has led many to conclude that environmental risk factors, such as a diet high in processed foods and low levels of physical activity, interact with genetic variation to shape NCD risk [4,5]. In other words, genetic variation may predispose individuals toward physiological sensitivity or resilience in the face of environmental perturbations, a phenomenon known as “genotype by environment” (GxE) interactions.

Despite major interest in GxE interactions in the context of NCDs, scientists have struggled in practice to identify them. There are many reasons for this, including that the relevant environmental factors are often unknown, difficult to measure, or minimally variable within the study population (e.g., most individuals in postindustrial contexts consume processed foods). Further, large sample sizes are needed to test for interaction effects, and even more so to overcome the multiple testing burden incurred by testing for interactions between many genetic variants and many environments [6,7]. To overcome power issues, current state-of-the-art approaches have leveraged very large studies such as the UK Biobank to scan for interactions between genome-wide genetic variation and selected lifestyle factors (e.g., smoking, diet, or physical activity) [8–11]. However, these studies have not delivered as expected and have only uncovered a handful of GxE interactions for NCDs such as obesity, type 2 diabetes, and depression.

In this Essay, we argue for a complementary approach informed by anthropological methods, genomic tools, and evolutionary theory. In particular, we believe there is much to learn by viewing GxE interactions through the lens of the “evolutionary mismatch” hypothesis and by partnering with genetically and environmentally diverse small-scale, subsistence-level populations to map them. The evolutionary mismatch hypothesis posits that traits that evolved under past selection regimes are often imperfectly or inadequately suited to modern environments, leading to “mismatches” in the form of NCDs [12–16]. At the genetic level, we would thus expect that previously neutral or beneficial alleles are now disease causing.

**Fig 2:**
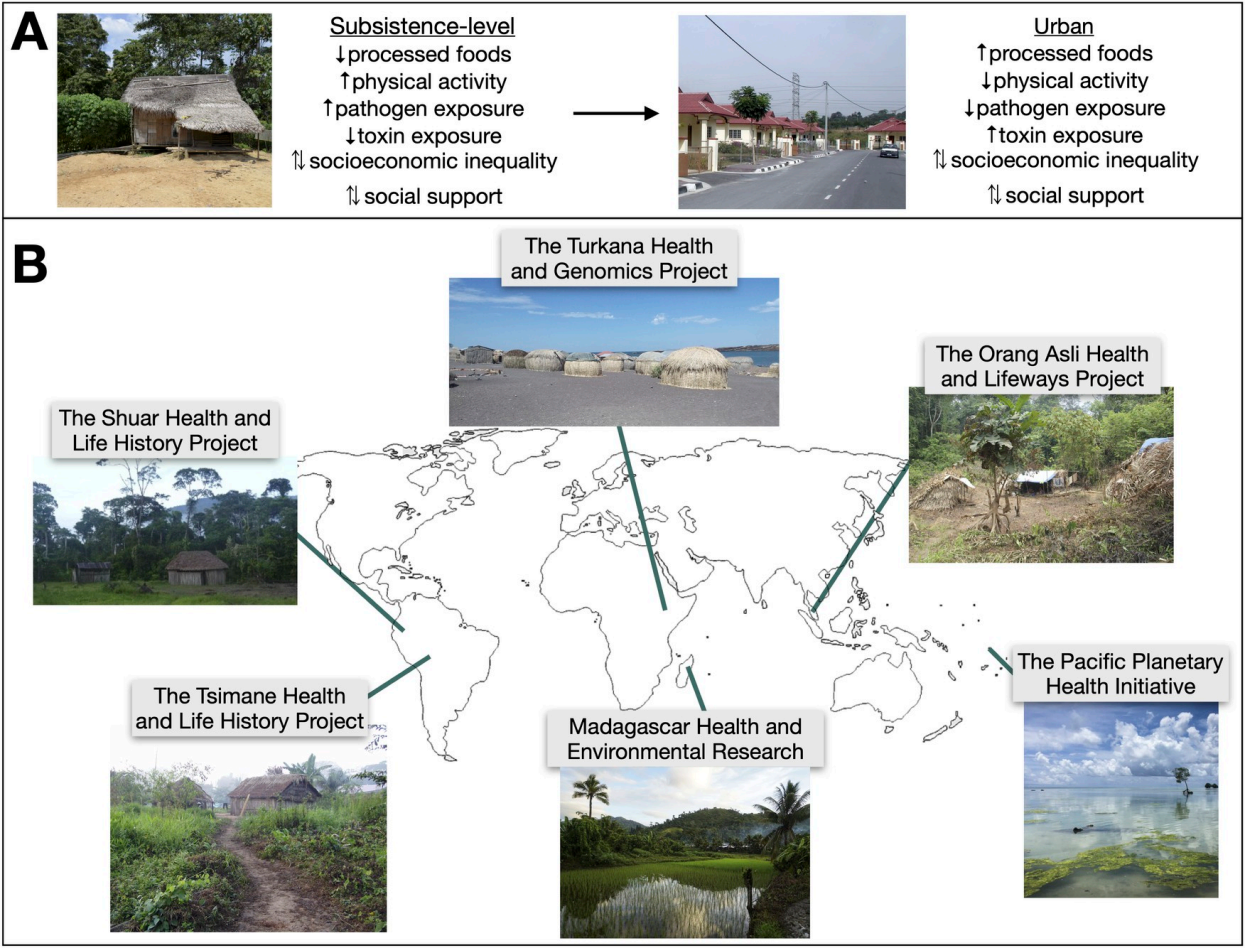
Subsistence-level groups experiencing lifestyle change are a potential model for uncovering GxE interactions. (**A**) Subsistence-level groups faced with urbanization, market integration, and modernization experience extreme variation in diet and physical activity levels, pathogen and toxin exposures, and social conditions. This list of environmental components for which there is extreme variation is not exhaustive and, in many cases, will also be population specific. We highlight a few broad categories that tend to change consistently during lifestyle transitions. Bidirectional arrows indicate factors that could either increase or decrease during urban transitions. (**B**) Studies such as The Turkana Health and Genomics Project [18,19], The Orang Asli Health and Lifeways Project [20], The Pacific Planetary Health Initiative, Madagascar Health and Environmental Research [21–23], The Tsimane Health and Life History Project [24], and The Shuar Health and Life History Project [25,26] all combine anthropological and biomedical data collection in transitioning societies and are thus poised to uncover GxE interactions in the context of evolutionary mismatch. We note that this list is meant to be illustrative and only includes projects directed by authors of this Essay; it does not by any means cover all of the rich and ongoing projects of small-scale, subsistence-level groups.

While we cannot go back in time to evaluate genotype–phenotype relationships in past environments, we can collaborate with populations that practice nonindustrial, subsistence-level lifestyles and thus fall further toward the “matched” end of the matched–mismatched spectrum than individuals in postindustrial contexts (though we caution that, of course, no modern population is perfectly representative of their evolutionary past). Further, many subsistence-level populations are currently exposed to globalizing forces causing rapid environmental shifts; this situation creates a quasi-natural experiment for studying the transition from traditional to modern lifeways within a single group [17] (Fig 2A). Additionally, the ecology and culture of many subsistence-level groups has already been well characterized through long-term work with anthropologists (Fig 2B), setting the stage for integration of genomic studies.

Uniting an evolutionary mismatch framework, long-term anthropological work with subsistence-level groups, and cutting-edge genomic tools can increase our power to identify and understand GxE interactions. Specifically, because the mismatch framework provides clear expectations for the types of loci and environments we expect to affect NCDs, we can narrow the search space considerably. Further, by focusing on populations where Western diets and lifestyles are the exception rather than the norm, we can design studies that explicitly sample environmental extremes, thereby boosting power. Finally, by studying many genetically distinct populations under a uniting intellectual framework, we can identify new loci that have so far been invisible to studies focused on individuals of European descent. With these goals in mind, we first review the evolutionary mismatch hypothesis and discuss its current support at the phenotypic and genetic levels. Second, we propose recommendations for integrating mismatch principles with molecular and genomic techniques, focusing on collaborations with subsistence-level groups. Third, we discuss the payoffs for scientists and study communities that would come from implementing these partnerships.

## Overview of the evolutionary mismatch hypothesis

An evolutionary mismatch is a condition that is more common or severe in an organism because it is imperfectly or inadequately adapted to a novel environment [27]. While mismatches are not unique to humans, their frequency may be unusually high in our species. This is because human culture can generate rapid and profound environmental change: In just a few generations, industrialization has transformed human diets, physical activity patterns, and toxin exposure landscapes, and these changes presumably contribute to the long list of NCDs that used to be rare or nonexistent [28–30].

**Fig 3:**
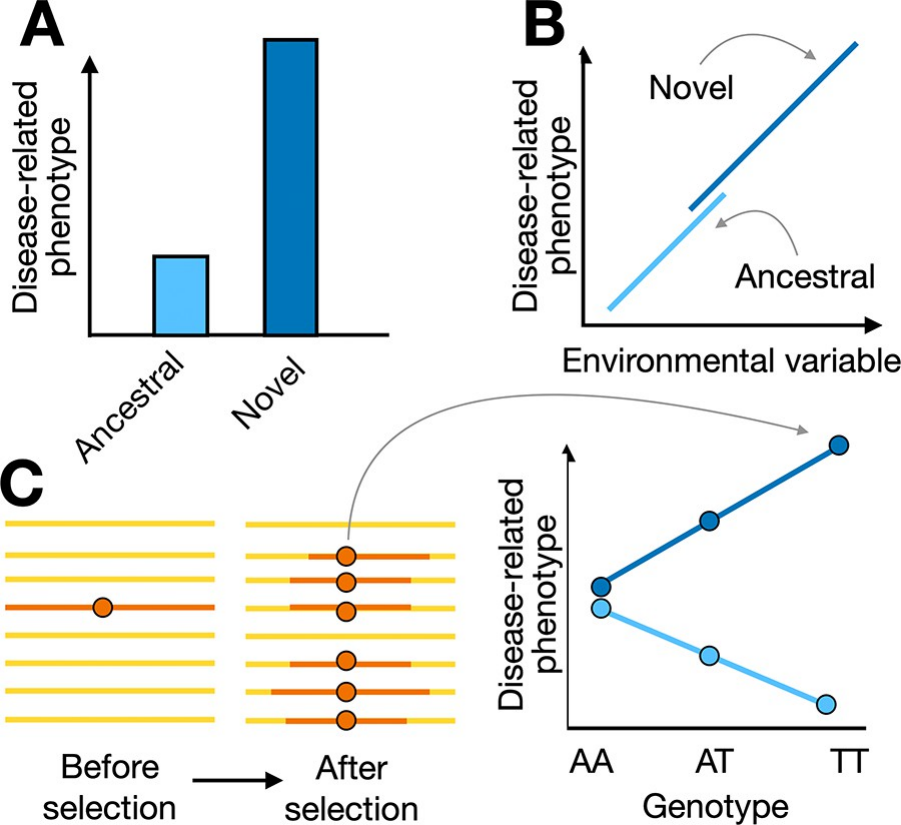
Mismatch diseases must be tested according to 3 criteria. (**A**) Disease-related phenotypes must be more common or severe in the novel versus ancestral environment. We note that here we show mean differences in the phenotype between environments, but environmental effects could also impact trait variance. (**B**) These disease-related phenotypes must be attributable to an environmental variable, which will most often differ in mean and range between groups (e.g., physical activity influences cardiovascular health and is consistently higher in subsistence-level groups relative to individuals in postindustrial contexts). (**C**) It is necessary to establish a mechanism by which an environmental shift generates variation in disease-related phenotypes. At the genetic level, this could manifest as a locus for which a variant exhibits a past history of positive selection and is associated with health benefits in the ancestral environment but health detriments in the novel environment. A single locus with opposing effects is shown here for simplicity, but in reality, most complex traits will have highly polygenic architectures and diverse patterns of GxE interactions [34]. In panel C, horizontal lines represent haplotypes and the dark orange circle represents the selected variant. In all panels, dark blue represents the novel environment and light blue represents the ancestral environment.

For at least a century, a wide range of conditions have been assumed to be “diseases of civilization” or “lifestyle diseases” [31,32], but mismatches need to be explicitly and rigorously tested according to 3 criteria [33]. First, a mismatch condition should be more common or severe in the “novel” (e.g., postindustrial) relative to the “ancestral” environment (or correlated with some continuous metric of novel versus ancestral; Fig 3A). Small-scale, subsistence-level societies typically stand in as the best available proxy for the “ancestral” environment. This is because modern subsistence-level societies on average experience a closer “match” between their recent evolutionary history and their current environments relative to individuals in postindustrial contexts, though we caution they are not themselves “ancestral” populations. We also caution that modern subsistence-level groups (or any human group) will never be perfectly matched to their ancestral conditions on any time scale, given the near-constant fluctuations in human cultures, ecologies, and lifestyles. Nevertheless, these populations do all experience major environmental components consistent with the human evolutionary past, namely, they eat diets devoid of, or low in, processed foods, engage in high levels of physical activity, and never or rarely experience medical intervention.

In addition to the hypothesized mismatch condition being more prevalent in postindustrial versus subsistence-level groups, the second criterion is that it should also be tied to some environmental variable that differs between these groups (Fig 3B). One complication for achieving this is that NCDs arise from complex multifactorial causes, and thus, while between-population comparisons are necessary, they can be confounded by many covariates that must also be taken into account (e.g., sanitation, access to medical care, or age structure, given that risk for most NCDs increases with age in postindustrial contexts [35]).

The third criterion is that it is necessary to establish a molecular or physiological mechanism by which the environmental shift generates the proposed mismatch condition. At the genetic level, this should manifest as a locus for which a variant exhibits a past history of positive selection and is associated with health benefits in the ancestral environment but health detriments in the novel environment, or one for which past stabilizing selection has created a situation where 2 intermediate alleles have similar fitness in the ancestral environment, but one allele becomes associated with health detriments in the novel environment (Fig 3C and Box 1).

**Box 1. GxE interactions in population genetics: Definitions and related concepts**

In population genetics, the simplest conceptualization of a GxE interaction involves 3 genotypes for a single biallelic locus, with each of the 3 genotypes found in 2 different environments and with fitnesses varying across these 6 conditions (Fig 3C). At equilibrium, this population will harbor, among other types of genetic variation, alleles that have been selected to high frequency as a consequence of directional selection (i.e., selection on a trait value in a particular direction), and alleles that are at intermediate frequency as a consequence of stabilizing selection (i.e., selection to keep trait values near an optimum). If the environment changes quickly, previously selected alleles may now be associated with a trait that is no longer beneficial, and even disease causing, but will remain at a high frequency for some time before selection is able to purge them.

A few notes are important on this simple thought example. First, loci with no genetic variation (e.g., fixed beneficial mutations) could still be involved in mismatches in the new environment, but in the absence of genetic variation, we will be unable to identify them. Second, most complex traits have highly polygenic architectures, and while our simple examples (here and throughout) have focused on a single biallelic locus, the same logic applies under polygenicity [36]. Third, stabilizing selection is thought to be the most common mode of evolution shaping complex traits [37], and, thus, mismatch scenarios involving alleles that have previously undergone stabilizing selection may be the most common.

In addition to GxE interactions, a quantitative genetic concept relevant to evolutionary mismatch is “decanalization” [16,38]. Canalization refers to the process of stabilizing selection that selects for trait values that closely track some optimum in a given environment. However, in the presence of rapid environmental change or other strong perturbations, the optimum can shift and lead to decanalization [39]. While canalization acts to decrease genetic and phenotypic variance in a trait over time, decanalization involves an increase in the trait’s variance that is generally thought to be associated with the unmasking of loci that only impact the trait in the new environment [40]. Decanalization can thus be thought of as a specific form of evolutionary mismatch. Evolutionary mismatch can occur without having a previously canalized trait and is a more general term not necessarily linked to stabilizing selection. A final term that is distinct from all of these is “robustness.” Robustness refers to a property of individual genotypes, wherein they are able to retain an advantageous phenotype despite genetic or environmental hazards [39]. In contrast, evolutionary mismatch and decanalization are population-level phenomena.

## Current evidence for evolutionary mismatch at the phenotypic level

**Fig 4:**
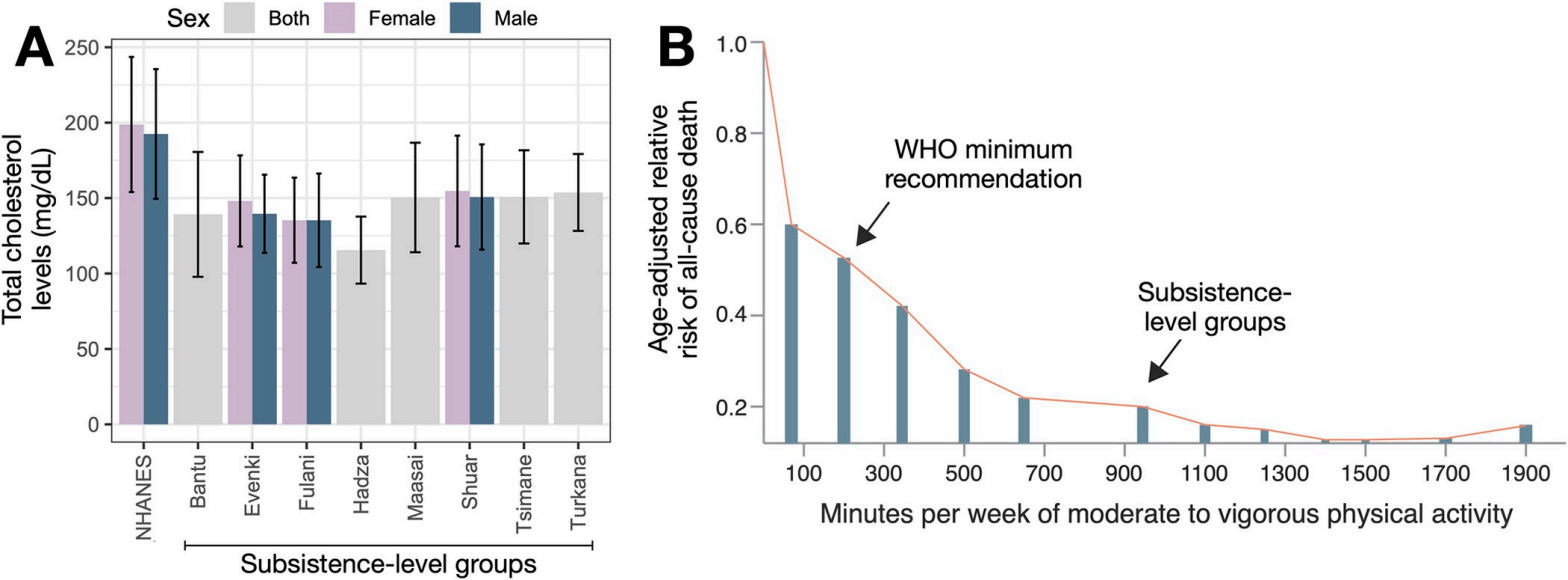
Evidence for evolutionary mismatch at the phenotypic level. (**A**) Mean levels of total cholesterol are much lower in selected subsistence-level populations relative to adults in the USA (>18 years old) profiled as part of the US National Health and Nutrition Examination Survey (NHANES) [49] (subsistence-level data from [17]). (**B**) Evidence that, within industrialized populations, individuals accruing daily physical activity similar to those of men and women in subsistence-level societies experience similarly low rates of CVD as well as all-cause mortality from NCDs. Dose–response relationship between minutes/week of moderate to vigorous leisure time physical activity and age-adjusted relative risk of death from a sample of 661,137 adults from the USA and Europe [50]. The arrow for physical activity estimates in subsistence-level groups is based on studies of Hadza individuals (estimated at x = 944 minutes [43]) and Tsimane individuals (x = 924 minutes [51]).

Scientists have been relatively successful at testing the first 2 criteria for mismatch, especially in the context of CVD, the single largest cause of mortality worldwide [41]. In support of the first criteria, subsistence-level groups experience remarkably low rates of CVD [30,42,43] relative to individuals in postindustrial contexts, as well as minimal age-associated increases in CVD or its biomarkers (e.g., hypertension, cholesterol) [44–46] (Fig 4A). Studies of small-scale societies in the midst of socioeconomic transition have demonstrated within-population effects of industrialization [18,47,48], strengthening the findings from between-population comparisons.

In support of the second criteria, recent work has also isolated salient environmental changes by which industrialization promotes CVD. People in subsistence-level communities are generally very physically active, accruing 5 to 10 times more daily physical activity than adults in postindustrial contexts [52,53]. Moderate to vigorous physical activity increases nitric oxide production and arterial elasticity [54,55] and reduces inflammation, all of which are protective against CVD [56]. Within industrialized populations, individuals accruing daily physical activity similar to those of subsistence-level individuals experience similarly low rates of CVD, as well as NCD-related mortality [57] (Fig 4B). Although physical activity has a critical role in averting CVD, it is not a panacea and several other factors are surely important. For example, relative to individuals in postindustrial contexts, subsistence-level groups subsist on diets dominated by unprocessed or minimally processed foods and experience different types and degrees of social integration and inequality, all of which can impact CVD risk [58–60].

We note that while we have focused this section on CVD as an illustrative example of the type of comprehensive evidence required for fulfilling the first 2 criteria of mismatch, several other conditions also have relatively clear evidence. For example, inflammatory and autoimmune disorders have increased during the 20th century, which has been linked to a reduced exposure to parasites and microorganisms (a phenomenon attributed to the “hygiene hypothesis” or “old friends hypothesis”) [61–63].

## Current evidence for evolutionary mismatch at the genetic level

As mentioned above, to fulfill the third criteria for mismatch, we would need to identify a locus for which there is evidence of past selection (positive or stabilizing), and for which performance of at least 1 allele varies across environments and confers inflated risk of an NCD in the novel environment (Fig 1B and Box 1). One would think this would be easy to find, but in fact, there are only a handful of clear cases, despite good evidence for the existence of GxE interactions in general [64–67]. One clear example of mismatch involves variants in *APOL1*, which provide resistance to trypanosome infections. Given the prevalence of trypanosomes across Africa, beneficial alleles are found at high frequency in African populations, as well as in African Americans. However, these same variants confer an increased risk of kidney disease in African American individuals living in the USA [68,69].

Another example is related to the “thrifty genotype” hypothesis [68], which suggests that individuals living in environments where food is unpredictably and periodically scarce should experience selection to store body fat in times of plenty. Recently, an intriguing variant was found in Samoans, who are also susceptible to extreme obesity when eating a Western diet: A single amino acid variant (p.Arg475Gln) in *CREBRF* exhibits signatures of past selection and is currently associated with a 1.3-fold increased risk of obesity (though puzzlingly, also a 1.6-fold decreased risk of type 2 diabetes). Subsequent functional work in cell culture models demonstrated that p.Arg475Gln has direct effects on metabolism, reducing energy use while increasing lipid storage [70].

In addition to these well-characterized examples (see also Fig 2 of [71]), recent genomic work has shown that, in aggregate, variants that serve as modern-day risk alleles for particular NCDs (namely, CVD and autoimmune diseases) are more likely to show signatures of past selection relative to nonrisk alleles [72–74]. More broadly, there is now ample evidence that human populations can adapt to their local ecologies quite quickly (e.g., in thousands of years) [75], setting the stage for mismatches when local conditions shift. For example, the high *Plasmodium vivax* malaria risk experienced by West Africans has selected for changes to a key chemokine receptor encoded by *DARC* [76,77], whereas the spread of dairying in Europe has selected for lactase persistence through changes in the regulation of *LCT* [78,79]. Both of these changes have occurred within the last 10,000 years. As pathogen environments and diets inevitably change, local adaptation sets the stage for mismatches to occur.

## A new path forward: Integrating genomic tools and partnerships with transitioning populations

In principle, GxE interactions are most simply identifiable using a mismatch framework by testing for environmentally dependent genetic effects in transitioning populations. However, in practice, this would be difficult because most NCDs arise from many small genetic effects distributed across the genome. Further, the standard approach to resolve this needle-in-a-haystack problem—using a massive sample size—is difficult in small-scale groups who typically have modest population sizes. Sample sizes in the thousands, but not hundreds of thousands (e.g., biobank scale), are currently feasible; however, many anthropological studies have invested in long-term relationships with particular communities and are thus able to generate highly longitudinal datasets [24], where repeated samples and within-individual study designs could boost power. With these limitations in mind, we discuss how advanced genomic methods can be combined with the mismatch framework in a principled way to quantify the role of GxE interactions in NCDs.

First, we can improve GxE test power by focusing on genetic loci with already demonstrated evidence for phenotypic relevance, for example, those with evidence for recent selection in the study group or those that have already been discovered in urban/industrialized environments. For example, recent work on the *APOE* locus found that the *E4* variant—a well-known risk factor for CVD and Alzheimer’s disease in individuals in postindustrial contexts—is associated with lower innate inflammation and may have beneficial effects on lipid moderation and cognition in a high pathogen/low obesity environment [80–82]. We might expect similar successes in elucidating GxE mismatches at other well-known risk loci that replicate across postindustrial contexts (e.g., *FTO*, *ADCY3*, *BRCA1/2*), though we caution that candidate gene studies should always be undertaken with care due to potential bias and replication issues [83,84]. A related approach is to test for GxE enrichment at the level of known genes or pathways with evolutionary or phenotypic relevance in the study population. These set-based approaches (i.e., that target predefined genes, genomic regions, or single nucleotide polymorphisms (SNPs)) may also perform well, even in cases where the specific causal variants are not shared between the focal population and the dataset in which they were identified.

Second, polygenic approaches that integrate GxE signals across the genome can improve power when studying complex traits such as NCDs. For example, recent methodological developments have extended the popular polygenic risk score (PRS) framework to allow for PRS–environment interaction tests, thus providing a polygenic GxE test [85–87]. This approach has so far been used to show how diet and other lifestyle factors modulate the genetic risk of obesity, metabolic traits, and type 2 diabetes [40,88–90]. While polygenic approaches such as the PRS sacrifice variant-level resolution, they yield much greater power to detect GxE interactions, an invaluable exchange for quantifying evolutionary mismatch in transitioning populations. Three downsides to PRS–environment interaction tests, however, are that compared to single, large-effect allele results, one can be left with no suggestion of underlying mechanism; power depends on the predictive power of the PRS as well as its portability, which is a clear problem, given that most PRS work has focused on European ancestry individuals in postindustrial contexts, and, thus, this is where the summary statistics to build a PRS in other groups will have to come from (for the time being); and an underlying assumption is that risk effects are systematically stronger in one environment than another [91]. Any work in this area will consequently require replication across populations and will dramatically benefit from biobank-scale datasets that are currently being built in underrepresented, non-European ancestry contexts (e.g., [92,93]); these datasets will surely catalyze better multiancestry PRS methods.

Third, and perhaps most feasibly with current sample sizes, we can add power and interpretability for GxE interactions using intermediate molecular phenotypes such as gene expression, DNA methylation, and chromatin accessibility. One approach is to impute these functional genomic features from genotype data and then test them for environmental interaction (e.g., akin to a GxE version of transcriptome-wide association studies) [94,95]. The imputation step can use large, publicly available functional genomic datasets from US and European cohorts but will improve when similar datasets are available for the study populations. A second approach is to directly measure gene expression, DNA methylation, or other molecular features and identify variants that impact these features in different ways across different environmental contexts; this “molecular QTL” framework has so far proven very powerful and could be extended to transitioning populations [64,96–98]. Moreover, GxE molecular QTLs can be validated experimentally by exposing cell lines or model organisms to stimuli that mimic aspects of the environmental gradients experienced by transitioning populations; indeed, this can pinpoint key components of the incredibly complex environmental shifts that drive GxE interactions. A third option is to use functional genomic experiments to narrow the search space, by first identifying regulatory elements that respond to mismatch-relevant environments. For example, Garske and colleagues recently identified chromatin elements that respond to dietary fatty acids in adipocytes and then focused GxE follow-up work on variants in these responsive elements. By doing so, they were able to gain power to search for interaction effects between genotype and dietary saturated fat intake on body mass index [99]. Similar in vitro functional genomic experiments (using field-collected samples) could be leveraged to target regions of the genome that may be most important for responding to key aspects of lifestyle transitions.

## Payoffs for NCD prevention and treatment

Testing the degree to which GxE interactions arise from evolutionary mismatch would answer mechanistic questions about how GxE interactions manifest. For example, are loci that were involved in adaptation to a population’s past environment more likely to exhibit GxE effects when the environment shifts? To what degree does the nature of GxE interactions vary across ancestries with distinct evolutionary histories? What is the envelope of “optimal” human environmental conditions that do not provoke mismatch? Molecular insights into evolutionary mismatch would allow us to prioritize the study of genetic variants that may adversely affect health outcomes in novel environments (i.e., those that have historically been under stabilizing or positive selection). It would also enable prediction of potential future adverse environments that could accelerate the onset of disease (i.e., those that represent strong deviations from the human evolutionary past). Furthermore, it could help us refine explanations for already observed ancestry-related differences in disease susceptibility. We emphasize that these are potential outcomes if mismatch is rigorously tested according to the criteria we lay out and subsequently supported; currently, its generalizability to the study of many complex traits and NCDs remains unclear due to a need for more empirical data.

The studies we recommend would more broadly advance our understanding of health issues in minority, Indigenous, and other underrepresented groups. Most subsistence-level populations in low- and middle-income countries (LMICs) are facing rapid rises in NCD risk, and the limited reports from these countries suggest that population responses to urbanization and market integration are highly variable. Studies of European ancestry individuals in postindustrial contexts are not well suited to explain why. Partnering with transitioning groups to conduct evolutionarily and culturally informed studies is needed to better serve their health concerns (Box 2).

**Box 2. Ethical considerations of conducting genomic work with subsistence-level populations**

Community engagement and ethical research is fundamental to achieving the broader vision of this Essay. There is widespread consensus that broader population representation in biomedical research is critical for reducing health disparities [100], but moving forward on this agenda requires that we simultaneously acknowledge and learn from past mistakes and abuses.

At the heart of ethical considerations in genetics research is a situation in which diverse populations are dually underrepresented and underconsulted [101]. Recent work has outlined best practices for overcoming these issues [101–108]. For example, Claw and colleagues [102] suggest 6 principles of research ethics: understand community sovereignty and research regulations; engage and collaborate; build cultural competencies; improve transparency; build local research capacity; and disseminate research in accessible formats. The common thread behind these principles is the importance of building trustful and long-term relationships based on principles of dynamic consent, reciprocity, beneficence, and sovereignty. In our own experience, building these sorts of relationships takes time (typically years) but is essential to do before engaging in research.

Basic research with populations in LMICs can lead to important insights, yet the value-added benefits from basic research (e.g., shaping health policy based on epidemiological trends, and/or the development of novel treatment strategies) often can take decades to materialize. Mechanisms for participant community involvement in these longer-term benefits should be explicitly embedded in initial plans [100]. It is also important to recognize that community benefits can extend beyond the research itself. The needs and desires of local communities will vary widely, but populations in LMICs may face problems that are deeply interconnected and often stem from systemic discrimination: poor nutrition and sanitation (often due to environmental degradation), minimal access to education, few economic opportunities, and loss of land rights. The priorities of communities will seldom match perfectly with the aims of scientists, especially when participant communities lack basic infrastructure and face discrimination. Prioritizing solutions to these problems is an opportunity to have great impact that will require cooperation between researchers, study participants, universities, nongovernmental organizations, governments, and funding bodies.

## Conclusions and future directions

The basic argument of this Essay is that we can further our understanding of evolution as well as the genetic architecture of human disease by combining genomic tools with studies of transitioning populations (as has been discussed previously [6,12,13,15,105,106], though not in the context of genomics). This recommended path improves on current approaches, which typically rely on “brute forcing” GxE scans across many SNPs and many environments. Instead, we advocate for using evolutionary theory to parse a priori which genotypes and environments we expect to interact. More specifically, under a mismatch framework, we expect genomic regions under positive or stabilizing selection in past environments to be enriched for GxE interactions revealed in postindustrial environments. If this framework proves true, leveraging its predictions could boost power and better position us to understand and predict GxE interactions in the etiology of NCDs. More generally, the work we propose would provide much needed insight into urgent health issues affecting vulnerable populations around the world.

Because the interdisciplinary perspective we take here necessarily touches on several fields, we did not attempt an exhaustive review of research on either evolutionary mismatch or GxE interactions (instead, we refer readers to excellent existing work [6,12,13,15,109,110]). However, there are several interesting new directions in these fields that are worth highlighting. For example, a growing body of work has begun to conceptualize the human microbiome as an evolved trait that is currently “mismatched” to its environment, often with serious health implications [111]. Given that the microbiome is under host genetic control and can therefore be a target of natural selection [112] and that industrialization can induce large scale changes in gut microbial communities [113–115], this is an exciting area in which to investigate GxE interactions that generate mismatch diseases. Another emerging research topic is sex differences in the response to lifestyle change: Several recent studies have found that women experience greater NCD risk following economic and nutritional transitions than men [18,25,116,117], yet how sex-specific genetic, physiological, or environmental variation interact to produce this phenomenon is still unknown [34]. Moreover, it is well established that early life experiences are important for predicting NCD risk later in life [118–120], and the timing of lifestyle change, as well as the degree to which individuals experience environmental mismatches within their lifetimes, may therefore be important to consider and to intersect with GxE frameworks (Box 3). In many cases, long-term partnerships with focal communities have already led to the creation of longitudinal datasets well positioned to take a life course approach. Moving forward, we expect that longitudinal perspectives on environmental change, NCD risk, and GxE interactions will be especially fruitful.

**Box 3. Life course perspectives on NCD risk**

Development is a period of heightened environmental sensitivity, and challenging experiences early in life increase lifelong risk of most NCDs [118,120,121]. Subsistence-level societies are an underutilized yet potentially powerful model for studying early life influences on NCD risk. Many of these groups are currently experiencing rapid lifestyle changes leading to (1) extreme variation in early life conditions within a single population, and (2) frequent mismatch between early life and adult environments—a situation that is thought to put individuals at risk for later life health issues [118–120]. Point 1 provides a clear opportunity to leverage the distributional extremes to study early life effects on health [26,122]. Further, point 2 affords us the opportunity to compare outcomes when individuals experience within-lifetime environmental “matches” versus “mismatches.” To date, studies of industrial transitions have come to mixed conclusions about the importance of within-lifetime mismatches [18,47,123,124]. More work in this area is urgently needed to understand when, why, and how early life experiences shape adult health in these groups.

Genomic tools applied to populations undergoing lifestyle change could also provide valuable insight into how early life experiences become “embedded” into lifelong physiology. At the molecular level, this process is thought to be mediated by stable changes in gene regulation (e.g., DNA methylation, chromatin accessibility, and gene expression). However, many gene regulatory elements are also dynamic and responsive to environmental perturbations throughout life. This fact leads to challenges in disentangling the effects of early versus later life environments, especially when the two are highly correlated (as is common in postindustrial contexts). By contrast, subsistence-level groups in transition often experience decoupled early life and adult experiences, which could be leveraged to disentangle early versus later life influences. Genotype data collected for the same individuals could also be used to identify rarely studied GxE interactions where the “E” encompasses early life experiences. Overall, integrative studies of transitioning populations are primed to reveal which individuals will be most susceptible to NCDs during lifestyle transitions as well as when in the life course these exposures matter most.

## Abbreviations

CVD: cardiovascular disease
GxE: genotype by environment
LMIC: low–and middle–income country
NCD: noncommunicable disease
PRS: polygenic risk score
SNP: single nucleotide polymorphism
