# Correction: Applying an evolutionary mismatch framework to understand disease susceptibility

**DOI:** 10.1371/journal.pbio.3003465

**Published:** 2025-10-29

**Authors:** The PLOS Biology Staff

In the Overview of the evolutionary mismatch hypothesis section there is an error in Box 1. Please see the correct Box 1 here:

**Box 1. GxE interactions in population genetics: Definitions and related concepts**.In population genetics, the simplest conceptualization of a GxE interaction involves 3 genotypes for a single biallelic locus, with each of the 3 genotypes found in 2 different environments and with fitnesses varying across these 6 conditions (Fig 3C). At equilibrium, this population will harbor, among other types of genetic variation, alleles that have been selected to high frequency as a consequence of directional selection (i.e., selection on a trait value in a particular direction), and alleles that are at intermediate frequency as a consequence of stabilizing selection (i.e., selection to keep trait values near an optimum). If the environment changes quickly, previously selected alleles may now be associated with a trait that is no longer beneficial, and even disease causing, but will remain at a high frequency for some time before selection is able to purge them.

A few notes are important on this simple thought example. First, loci with no genetic variation (e.g., fixed beneficial mutations) could still be involved in mismatches in the new environment, but in the absence of genetic variation, we will be unable to identify them. Second, most complex traits have highly polygenic architectures, and while our simple examples (here and throughout) have focused on a single biallelic locus, the same logic applies under polygenicity [36]. Third, stabilizing selection is thought to be the most common mode of evolution shaping complex traits [37], and, thus, mismatch scenarios involving alleles that have previously undergone stabilizing selection may be the most common.

In addition to GxE interactions, a quantitative genetic concept relevant to evolutionary mismatch is “decanalization” [16,38]. Canalization refers to the process of stabilizing selection that selects for trait values that closely track some optimum in a given environment. However, in the presence of rapid environmental change or other strong perturbations, the optimum can shift and lead to decanalization [39]. While canalization acts to decrease genetic and phenotypic variance in a trait over time, decanalization involves an increase in the trait’s variance that is generally thought to be associated with the unmasking of loci that only impact the trait in the new environment [40]. Decanalization can thus be thought of as a specific form of evolutionary mismatch. Evolutionary mismatch can occur without having a previously canalized trait and is a more general term not necessarily linked to stabilizing selection. A final term that is distinct from all of these is “robustness.” Robustness refers to a property of individual genotypes, wherein they are able to retain an advantageous phenotype despite genetic or environmental hazards [39]. In contrast, evolutionary mismatch and decanalization are population-level phenomena.

The publisher apologizes for the errors.
